# Benefits of Using Fluorescence Induced Theragnosis in Fixed Orthodontic Therapy: Status, Technology and Future Trends

**DOI:** 10.3390/dj9080090

**Published:** 2021-08-05

**Authors:** Anand Marya, Liviu Steier, Mohmed Isaqali Karobari, Adith Venugopal

**Affiliations:** 1 Department of Orthodontics, Faculty of Dentistry, University of Puthisastra, Phnom Penh 12211, Cambodia; 2 Department of Orthodontics, Saveetha Institute of Medical and Technical Sciences, Saveetha Dental College, Saveetha University, Chennai 600077, India; avenugopal@puthisastra.edu.kh; 3 Department of Restorative Dentistry, University of Pennsylvania, Philadelphia, PA 19104, USA; lsteier@gmail.com; 4 Conservative Dentistry Unit, Health Campus, School of Dental Sciences, Universiti Sains Malaysia, Kubang Kerian, Kota Bharu 16150, Kelantan, Malaysia; dr.isaq@gmail.com

**Keywords:** biofilm, fluorescence, orthodontics, theragnosis

## Abstract

Dental biofilm is often found to be the source of bacteria that releases toxins, peptides, lipopolysaccharides as well as organic acids, which lead to gingival inflammation and tooth caries. Further, the persistent plaque may result in the continued destruction of the surrounding soft and hard tissues. During fixed orthodontic therapy, arch-wires, brackets, and elastic modules have been shown to be sites of significant plaque accumulation, making it difficult for a patient to maintain proper oral hygiene. The problem most dentists face is that they cannot visualize this biofilm completely to be able to carry out efficient plaque removal. Visual assessment is, to date, the most common method for plaque visualization, and various indexes have been demonstrated to be sufficient for quantification of the amount of plaque present. However, the problem is that visual assessments are inconsistent, operator dependent and often subjective, which can lead to inconsistency in results. Fluorescence is one such method that can be explored for its use in effective plaque identification and removal. Literature has it that dentists and patients find it particularly useful for monitoring oral hygiene status during treatment. Fluorescence has the capability of offering clinical orthodontists and researchers a new method of detection of demineralization during orthodontic treatment, furthermore, for efficient removal of orthodontic adhesive cements, fluorescent light may be used in conjunction with high-speed burs to deliver fast, less time consuming, and safer results. The benefit of direct visual treatment using fluorescence enhanced theragnosis is that the patient receives controlled and guided therapy. It has multiple benefits, such as early diagnosis of caries, biofilm identification, and even helps to achieve improved treatment outcomes by better resin selection for esthetic procedures.

## 1. Introduction

Dental plaque is formed by a variety of bacteria that form an adhesive layer on the tooth surface. These bacteria can accumulate rapidly and form colonies that lead to the development of a biofilm that collects in the interdental spaces and gingival margins [1,2]. Once the biofilm is formed, there is a release of toxins, peptides, lipo-polysaccharides, and organic acids. The accumulation of these products leads to gingival inflammation and tooth caries over time [3,4]. If this dental biofilm is removed in time, gingival health can be restored without any soft or hard tissue damage [5]. However, if the biofilm is allowed to persist and proliferate, it can damage the periodontal tissues and cause enamel caries [6,7]. With time, biofilm can even lead to the formation of periodontal pockets and move in a more sub-gingival direction because of the gradual development of a more complex non-aerobic local environment [8]. As long as the biofilm is present, there will be continued destruction of the surrounding soft and hard tissues. The problem most dentists face is that they cannot visualize this biofilm completely to be able to carry out efficient plaque removal. Orthodontists face an even more difficult problem as the presence of fixed attachments on teeth in brackets and wires makes it very complicated to estimate the plaque progression. They rely on the patients to maintain good oral hygiene during the treatment duration and can only carry out prophylaxis using disclosing solutions when they visit for a follow-up appointment [9]. 

During fixed orthodontic therapy, arch-wires, brackets, and elastic modules have been shown to be sites of significant plaque accumulation, making it difficult for a patient to maintain proper oral hygiene [10]. The prolonged presence of plaque can lead to demineralization in the 4-week interval between appointments [10]. At every follow-up appointment, a patient undergoing fixed orthodontic therapy must be clinically assessed for plaque accumulation, and the importance of good oral hygiene must be reinforced. Visual assessment is the most common method for plaque visualization, and various indexes have been demonstrated to be sufficient for quantification of the amount of plaque present [11]. While visual assessment is effective for plaque identification, it is not enough to visualize demineralization as considerable changes may occur below the surface before a white spot is visible [12]. Various studies conducted on oral hygiene reinforcement during orthodontic treatment have focused chiefly on analyzing periodontal health, techniques, and the quantification of plaque formation [13]. Research has also been conducted on improving oral hygiene in patients that have been positively reinforced with or without repeated oral hygiene instructions [14]. To motivate the patients, the first step has to be the visualization and assessment of the patient’s oral hygiene status. Various methods and interventions have been used to disclose agents, reward systems, report cards, and virtual reminders [15,16,17,18]. However, the problem is that visual assessments are inconsistent, operator dependent, and often subjective, which can lead to inconsistency in results. Methods such as disclosing solutions can lead to tissue staining and subsequent esthetic concerns, while the patients can always ignore reports and reminders.

**Figure 1:**
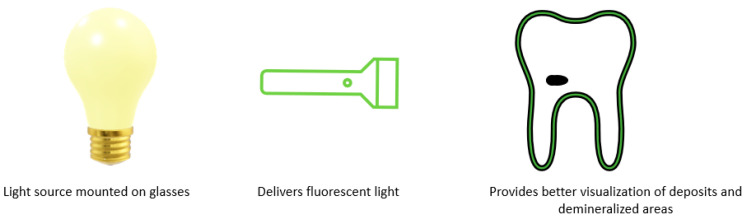
Graphical representation of how fluorescent light aids in visualization of deposits and demineralized areas. Different pathogenic bacteria within a biofilm produce porphyrins. Once excited with certain wavelengths these emit light in certain wavelengths. Special filters are needed by the viewer to correctly visualize the emitted light and diagnose bacterial presence. The wavelength of the exciting light has to fit into the spectrum of the targeted visualisation goal.

A non-invasive and objective technique to detect and assess plaque clearly would be of great value in orthodontic patients [19]. Fluorescence is one such method that can be explored for its use in effective plaque identification and removal (Figure 1). Fluorescence has been explained as a process where substances absorb a shorter wavelength of light and emit longer wavelengths. These substances contain molecules called fluorophores that undergo de-excitement from a higher to a lower energy state on light absorption [20,21]. Previously published literature has shown that dental plaque fluoresces when exposed to 405 nm light even without using a disclosing agent [22]. On further research, it was also seen that old and cariogenic plaque is more susceptible to fluorescence than plaque recently formed [23]. Once the plaque emits fluorescent light, it can be easily detected using a dedicated camera that allows easy visualization and effective removal. Fluorescence has been found to be very effective in detecting oral mucosal alterations and early prediction of oral cancers [24]. It has also been shown to be effective in defining bone-resection margins in surgically treated cases of osteonecrosis [25].

To understand the various benefits fluorescence can provide during fixed orthodontic therapy, various scenarios can be considered. This paper aimed to review existing literature related to fluorescence enhanced theragnosis and provide future uses and benefits of using this modality across various areas of orthodontic treatment. 

## 2. Hygiene Motivation and Monitoring during Treatment

**Figure 2:**
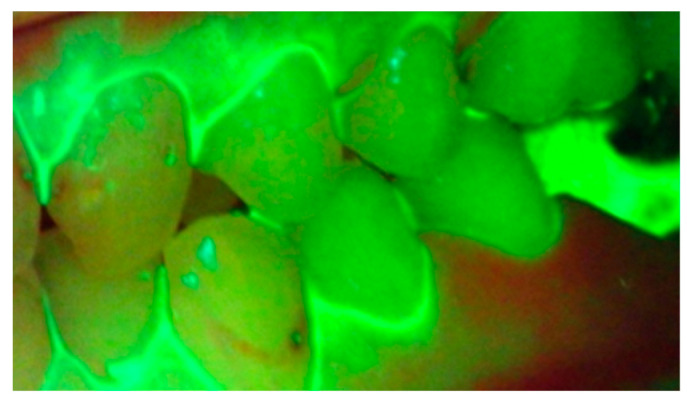
Oral hygiene and plaque accumulation status highlighted using fluorescent light.

It has been seen that the leading cause of periodontal problems and dental caries during fixed orthodontic therapy is the failure to remove plaque efficiently, effectively, and promptly. Any additional costs can be avoided during orthodontic therapy by providing an effective preventive solution to identify and remove plaque [26,27]. During the active stages of treatment, it has been seen that there is increased plaque accumulation because of the role of brackets and wires in protecting plaque from saliva, mastication, and brushing [28,29,30,31]. Since plaque in orthodontic patients cannot be highlighted easily, it becomes difficult to assess, educate, and motivate patients to improve their oral hygiene status. Studies conducted using fluorescence have shown that dentists and patients find it particularly useful for monitoring oral hygiene status during treatment [32]. It was seen in such studies that there was a reduction in plaque coverage on the brackets, highlighting the importance of regular oral hygiene reinforcement during fixed orthodontic treatment (Figure 2). Furthermore, images recorded with fluorescent light may prove beneficial for diagnosis and be a valuable addition to the patient records for progress, medico-legal purposes, and research. 

## 3. Early Demineralization Detection

**Figure 3:**
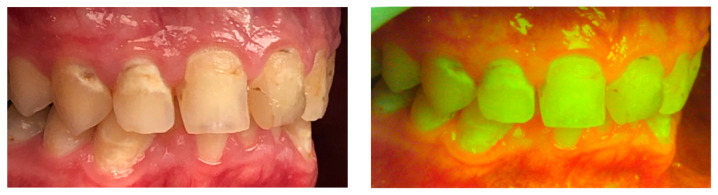
Comparison of intra-oral pictures depicting white spot lesions with and without fluorescent light.

Demineralization is one of the most commonly occurring white spot lesions during orthodontic treatment [33,34]. Not only do these cause esthetic concerns but also predispose the affected teeth to caries. Previous research has shown that the incidence of white spot lesions is two times higher in patients undergoing fixed orthodontic therapy [35]. The incidence increases further with the consumption of dietary items containing sugar [35]. An aim of early demineralization detection is that remineralization can be carried out to avoid any aesthetic damage or the need for restorations. The use of fluorescent light is an indirect method of estimating the demineralization process, which utilizes the relation between the fluorescence intensity of the enamel and the mineralization status [36] (Figure 3). Fluorescence has the capability of offering clinical orthodontists and researchers a new method of detection of demineralization during orthodontic treatment. 

## 4. Resin Cement Assessment and White Spot Lesions after Debonding

Previous research has confirmed the ability of fluorescence to measure white spot lesions and the presence of excess resin cement after debonding orthodontic brackets [37]. Teeth bonded with orthodontic brackets usually accumulate plaque and demonstrate white spot lesions of varying degrees when the treatment is completed, and the brackets removed [38]. Once the brackets are removed and the affected surface is exposed to saliva and brushing, these lesions are arrested, and remineralization can be carried out using fluoride-based agents [39]. Fluorescent light has been shown to be very effective in demonstrating white spot lesions as dark areas on images [40]. 

Even though bonding of orthodontic brackets is an established process, there are many challenges when it comes to removing the adhesive composite remnants [41]. Common problems encountered in removing the resin cement during debonding are varying degrees of accessibility, primarily in the molar regions, visualization, and the conditioning area required for different teeth [42]. For efficient removal of orthodontic adhesive cements, fluorescent light may be used in conjunction with high-speed burs to deliver fast, less time-consuming, and safer results. 

## 5. Comparison of Various Orthodontic Materials in Terms of Plaque Accumulation

If we can positively utilize digital technology to deliver better care to our patients, it is our responsibility to do so. This requires constant research and innovation, and the data collected for such studies must be reliable [41]. Conventional plaque estimation methods include dyes followed by a visual assessment which is error-prone, may demonstrate bias, and is both time and cost-consuming [43]. The use of light-induced fluorescence-based devices can reduce the errors associated with many of the conventional data collection methods. The ability to measure even subtle changes in the minerals contained in the tooth structure makes this a convenient tool for research purposes [43].

## 6. Fluorescence Enhanced Theragnosis—The Next Stage of a Targeted Diagnostic Approach?

**Figure 4:**
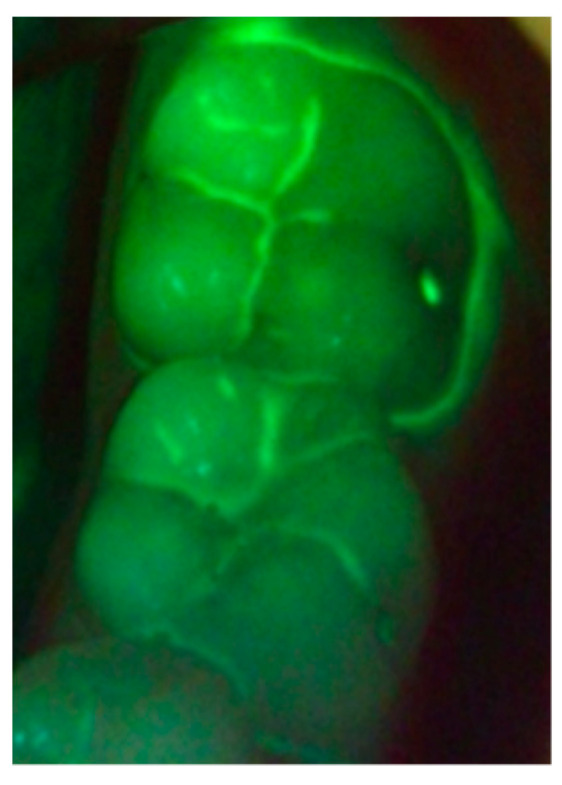
Definite and clear visualization of demineralized areas, caries, and plaque accumulation around the teeth using fluorescent light.

Daylight and magnification have both been long established as two pillars supporting oral diagnosis and treatment planning. Using a definite light spectrum, photoluminescence can be created, which can be visualized using specific filters. This is possible due to the inherent fluorescent properties of the tooth and porphyrins generated by caries causing bacteria. The benefit of direct visual treatment enhancement using theragnosis is that the patient receives controlled and guided therapy. Fluorescence enhanced theragnosis has multiple benefits, such as early diagnosis of caries, biofilm identification, and even helps achieve improved treatment outcomes by better resin selection for esthetic procedures (Figure 4). Fluorescence enhanced theragnosis for the diagnosis of demineralization is today more successful if accompanied by the use of external fluorophores with a direct focus on Ca ions. This does need more clinical trials to demonstrate its viability in clinical dentistry. Fluorescence enhanced theragnosis needs more attention and research to help incorporate this into a valuable diagnostic and treatment planning tool in orthodontics with such vast potential.

## 7. Areas of Improvement

Fluorescence enhanced theragnosis can be carried out using a wearable device called Reveal (Designs for Vision, Inc., Bohemia, NY, USA) [44]. This device consists of glasses with magnifying loupes, both coated with multiple protective layers to prevent any eye damage from the emitted light. With further use and mass production, the cost of these devices can be brought down to ensure wider use across the orthodontic community.

## 8. Conclusions

Since the advent of the digital era, orthodontists have been at the forefront of adopting various technological methods and techniques to help provide better treatment options to their patients. Aristotle said, “Well begun is half done,” and if we can ensure constant monitoring of treatment progress for a better outcome, we must take that responsibility. 

## Data Availability

Not applicable.
